# The Group Intertemporal Decision-Making Process

**DOI:** 10.3390/bs14090815

**Published:** 2024-09-14

**Authors:** Hong-Yue Sun, Yi-Ting Xiao, Shan-Shan Yang

**Affiliations:** Department of Psychology, Shanghai Normal University, Shanghai 200234, China; xiaoyt0640@163.com (Y.-T.X.); 1000495252@smail.shnu.edu.cn (S.-S.Y.)

**Keywords:** group intertemporal decision making, decision-making process, interpersonal interaction, information processing

## Abstract

Intertemporal decision making is the process by which individuals make judgments or choices regarding outcomes that occur at different times. Although intertemporal decision making is widely investigated, most studies explore it in terms of individual decision making, while neglecting group decision making, which holds more practical significance and adaptive value. This study recommends adopting a “two-process” approach that uses self-assessment questionnaires, audiovisual recordings, and visual decision-making tools to quantify interpersonal interaction processes and information processing in group intertemporal decision-making settings. In this way, studies can reveal the psychological and theoretical mechanisms of the group intertemporal decision-making process. At the level of interpersonal interaction processes, such an approach can identify the differential mechanisms between group and individual intertemporal decision making. In terms of information processing, it can reveal the mechanisms of the decision-making process in group intertemporal decision making. The findings of such work can provide a basis for interventions and nudges that encourage more visionary group intertemporal decision making.

## 1. Introduction

In intertemporal decision making, people make judgments or choices about outcomes that occur at different points in time [1]. Intertemporal decision making typically presents two types of options: one that offers smaller rewards that come sooner, and another that offers larger rewards that come later [2]. From individuals and families to societies and nations, intertemporal decision making has always been an important part of human decision-making behavior. Investors, for example, face the intertemporal decision problem of choosing between speculative behavior that meets short-term interests and investment behavior that satisfies long-term interests.

Although there is abundant research on intertemporal decision making, most studies focus on individual decision making, ignoring group decision making, which is a more realistic and adaptive form of decision making. Groups such as households, firms, and even countries make many intertemporal decisions. For example, family members might jointly determine the family’s savings plan, a firm’s directors decide upon the company’s investment plan, and governments make decisions about energy and environmental policy. In group decision making, multiple decision makers interact, influence each other, and share information, which culminates in the formation of a consensus preference within the group [3]. With its broader and more enduring implications for our daily lives, this type of decision making is distinguished from individual decision making. Groups tend to have more problem-solving resources than individuals, and the diversity of group resources can compensate for a lack of individual knowledge and skills [4,5]. For example, measures to contain COVID-19 were rapidly formulated after its outbreak in late 2019, through communication and negotiation among disease control experts, researchers, and local government officials [6].

Previous studies have shown that future returns are associated with greater uncertainty and risk compared with immediate returns, which increases the difficulty of decision making [7]. As a result, individuals often prefer immediate gratification and tend to opt for smaller immediate rewards, such as engaging in substance abuse, internet addiction, usury, or ecological destruction for short-term gains, which are manifestations of shortsightedness [8,9,10]. This shortsighted decision making can lead to a significant waste of individual and societal resources. In economics, for instance, myopic decisions at both the individual and policy levels can lead to overconsumption and debt accumulation, hindering long-term stability and growth and potentially triggering economic crises [11,12]. Regarding the environment, meanwhile, shortsighted individuals often do not focus on long-term benefits, making it difficult for them to adopt green behaviors [13]. In terms of personal health, individuals with “high temporal discounting” might prefer immediate satisfaction, such as choosing high-calorie foods while ignoring their long-term health risks [14]. Uncertainty about future outcomes was amplified during the COVID-19 pandemic, when many people were unsure about the efficacy of vaccines, the timing of lifting restrictions, and the resumption of work [15,16]. This tended to intensify people’s impatience, making them more inclined to seek immediate satisfaction, which sometimes manifested as interpersonal violence, impulsive shopping, or addiction [17]. Studies have confirmed that COVID-19 also affected individuals’ intertemporal economic choices, with people preferring immediate rewards more so during the pandemic than before it [18]. Individuals’ time discounting rates increased as the pandemic evolved, and then decreased after it was controlled [19].

In light of such phenomena, studies have focused on ways to effectively reduce the rate of temporal discounting [20,21,22,23], specifically by posing the following questions: (1) Can group intertemporal decision making help overcome the myopia of individual intertemporal decision making? (2) What are the differences between the mechanisms of group and individual intertemporal decision making? (3) What is the decision-making process in a group intertemporal decision-making setting, and (4) how can it be made more farsighted? Answering these questions can not only enrich our understanding of intertemporal decision-making theory, but also help guide decision-making practices, especially in the face of global crises, such as COVID-19, to foster more rational and sustainable decision making through collective wisdom.

## 2. Theoretical Development of Intertemporal Decision Making

Intertemporal decision-making research is traditionally based on the assumption of unbounded rationality, which assumes that individuals make decisions based on the principle of expected utility maximization. That is, after calculating the sum of utilities for all outcomes of each option, a choice is made to select the option with the greatest total utility. The discounted utility model [24] suggests that individuals discount the utility of future points in time at a constant rate, which is known as the delay discounting rate. A higher delay discounting rate indicates more impatience and a reduced willingness to wait for future benefits. Subsequent models of intertemporal decision making, such as the hyperbolic model [25] and the quasi-hyperbolic discounting model [26], despite their different functional forms, have not deviated from the principle of expected utility maximization.

Since the 1980s, however, with the rise of behavioral economics, experimental studies have found that people systematically violate the discounted utility model and exhibit “anomalies” [25,27,28,29,30,31]. This compelled researchers to refine models to fit data observed in reality. Various theories have aimed to demonstrate the applicability of the expected utility principle in intertemporal decision making by modifying discount functions (e.g., the hyperbolic discount function) or introducing new variables into the utility function (e.g., the expected utility function).

Nobel laureate Herbert Simon proposed the bounded rationality hypothesis, suggesting that decision makers, constrained by computational abilities and other factors, are not infinitely rational. Based on the assumption of bounded rationality, some researchers have proposed that, to meet survival needs, individuals apply simpler and more practical principles to decision making [32]. These researchers have found that the accuracy of heuristic methods surpasses that of complex strategies [33], leading to the abandonment of the expected utility principle and the establishment of other, more streamlined, decision-making theories. Among them, the single-dimension priority model is representative, including the tradeoff model [34] and the equate-to-differentiate theory [35]. The single-dimension priority model posits that, in intertemporal decision making, decision makers must compare options along the dimensions of delay and outcome, and then make choices based on the dominant dimension. If the difference in outcomes is greater than the difference in delays, the decision maker will make decisions based solely on the outcome dimension—that is, they will choose the option with the larger outcome. Conversely, if the difference in delays is greater than the difference in outcomes, the decision maker will make decisions based solely on the delay dimension—that is, they will choose the option that yields results sooner.

Meanwhile, psychological research has also provided explanations for intertemporal decision making from a cognitive perspective, such as dual-process theory. Dual-process theory posits that decisions are made by two cognitive systems: an intuitive and rapid System 1, which leans towards immediate gratification, and a deliberate System 2, which engages in more measured and future-oriented considerations [36]. This theory sheds light on explaining the “biases and anomalies” observed in real-life decision making. Some empirical studies, such as that of Costa et al. [37], have substantiated the existence of these two systems. Through the experiment of cooperative decision making under uncertainty, it has been found that, with an increase in cooperative frequency, individuals shift from intuitive decision making (System 1) to more deliberative decision making (System 2) [37]. Although dual-process theory has been questioned concerning its theoretical robustness and applicability [38], it is being refined and extended in the light of new experiences. Based on previous research, a dynamic dual-process model proposed by Diederich and Zhao [39] introduces a formalized approach to capture the stochastic elements and temporal dynamics of intertemporal choices. This model not only generates testable predictions about choice probabilities and response times, but also provides a theoretical bridge between dual-process theory and observable decision-related behaviors. In the future, a similar integrative approach that combines mathematical modeling with psychological analysis of behavioral decision making could further enhance the theoretical predictability and practical guidance of research on intertemporal decision making.

## 3. Group Intertemporal Decision Making

As mentioned earlier, current research on intertemporal decision making mostly focuses on individual decision making, while ignoring group decision making.

### 3.1. Influence of the Social Environment on Intertemporal Decision Making

Group intertemporal decision making is concerned with how individual decisions are influenced by others and the social environment. Many studies of intertemporal decision making have investigated how it is influenced by the social environment, providing a foundation for conducting intertemporal decision making in group interaction situations.

There are differences between self-decision making and decision making by others, which includes advising and acting on behalf of others. Irrational behavioral tendencies, such as choice overload and omission bias, occur more frequently in self-decision making, whereas decision making by others exhibits fewer violations of rational decision making principles [40,41,42]. Nonetheless, self-decision making is less likely to exhibit the compromise effect and predecisional distortion, which are types of decision biases [42]. Research on intertemporal decision making has likewise focused on the difference between making decisions for oneself and making decisions for others [43,44,45,46,47]. Albrecht et al. [43] found that participants with larger discounting rates were more likely to choose future options when making decisions for others, and the brain regions responsible for emotions showed higher levels of activation during self-decision making than during decision making for others. They also found that people made different intertemporal decisions when alone versus in the presence of others. Another study found that late adolescents were more likely to prefer immediate rewards to delayed rewards in the presence of a peer than when alone [48]. Observing a shortsighted peer in an intertemporal choice task can also make one more inclined to choose the immediate option [49,50]. Meanwhile, when an adult is present, adolescents tend to make intertemporal choices that are more biased toward long-term options [51].

### 3.2. Decision Outcomes of Group Intertemporal Decision Making

There is relatively little research on group intertemporal decision making. The limited existing studies have mainly examined the outcomes of group intertemporal decisions, exploring the differences between these outcomes and those of individual intertemporal decisions. They have also focused on the effect of decision makers and other factors on the results of group intertemporal decision making.

#### 3.2.1. Comparison of Group and Individual Intertemporal Decision-Making Outcomes

Research on the differences between group and individual intertemporal decision-making outcomes is still preliminary, and there is little research on the factors and mechanisms affecting these differences. Studies conducted tasks that included a pre-individual decision-making stage, a group decision-making stage, and a post-individual decision-making stage, identifying the “average effect” and “convergence effect” [51,52,53,54]. The average effect refers to the strong correlation between the delay discount rate of group members during the group decision-making process and the average individual delay discount rate of its members before forming the group. The convergence effect reflects the impact of cooperative experience in group decision making on subsequent individual decisions, indicating that, after the group decision-making stage, group members exhibit a discount rate closer to the group delay discount rate during the individual decision-making stage. Using a between-subjects design to compare group and individual intertemporal decision making, Liu [55] found that group intertemporal decision making tended to prefer the long-term options.

#### 3.2.2. Factors Influencing Group Intertemporal Decision-Making Outcomes

Studies have explored the effects of intertemporal decision-making preferences, social distance, and heterogeneous gains among group members on group intertemporal decision-making outcomes. Tsuruta and Inukai [56] explored the effects of members’ decision-making preferences in a three-person group on the group’s delay discounting rate. They found that, when the delay discounting rates of the three people in the group were distributed from high to low, the final group intertemporal decision was closer to the decision preference of the member with the middle-level delay discounting rate. That is, members with the highest and lowest delay discounting rates changed their decision preferences and converged to the member in the middle. Schwenke et al. [57], meanwhile, explored the effect of social distance among group members on the delay discounting rate in group intertemporal decision making. They found that the group of socially distant participants tended to choose the delay option more than the group of socially non-distant participants. This is because people tend to be reluctant to take more risks when making decisions for their friends because of high responsibility; when making decisions for strangers, however, responsibility is lower; hence, risk aversion is lower [58]. Therefore, members in socially distant groups choose the delay option due to lower risk aversion. Glätzle-Rützler et al. [59] explored the effects of heterogeneous gains among group members in group intertemporal decision making. The experiment was divided into two phases—individual intertemporal decision making and group intertemporal decision making—wherein each participant completed an individual decision before completing the group decision together, and the cost for the participant was randomly selected from either of the two phases. In the individual decision-making phase, there were two cases of delayed amount allocation for each member in the three-person group, which were high–high–low and low–low–high combinations. The high–high–low and low–low–high groups both preferred the delayed option compared with the individual decision-making phase. This was attributed to the dominant role played by the participants in the group who enjoyed high delayed payoffs, which influenced the choice preferences of the other members of the group, thus making the final group delay discounting rate lower, in favor of the delayed option.

### 3.3. Group Intertemporal Decision-Making Process

Group decision making can be understood as an information-processing process accompanied by interpersonal interactions [60,61]. Granovetter’s [62] behavioral contagion model suggests that group interactions can systematically change individual attitudes, making it possible for decision makers to change their initial preference structure. In group decision making, interpersonal interactions, such as stating opinions, persuading others, and negotiating, are common among members [61]. The form of interpersonal interaction indirectly affects the quality of group decision making by influencing the exchange and flow of information among group members, while the depth of information processing directly affects the quality of group decision making.

#### 3.3.1. Interpersonal Interactive Process

Bixter et al. [54] found that the hierarchical status of group members affects the process of intragroup interpersonal interactions and ultimately influences group intertemporal decision-making outcomes. The decision-making preferences of in-group leaders typically influence those of non-leaders and ultimately have stronger effects on group intertemporal decision-making outcomes. Moreover, non-leader members more strongly identify with leaders appointed by the experimenter, compared with randomly appointed leaders. Furthermore, leaders appointed by the experimenter have a stronger influence on non-leaders’ decision preferences. Since the social environment can also affect individual intertemporal decision-making outcomes, it is necessary to consider whether changes in group intertemporal decision-making outcomes are attributable to internal interpersonal interaction processes, or whether social environmental factors are at play. Schwenke et al. [63] proposed two factors that might affect group intertemporal decision-making outcomes: One is interchange in interpersonal interactions—that is, a process in which a group benefits from the exchange of multiple members’ perspectives, areas of specialization, information reserves, and cognitive resources [64]. The other is social facilitation—that is, the effect of the social environment itself on an individual’s behavioral performance, such as an individual’s desire to eat being more controlled when others are present [65]. Schwenke et al. [63] had a two-person team manipulate a cursor to complete a cross-phase choice task. The task was divided into three phases: the individual decision-making phase, the pre-decision-making phase, and the cooperative decision-making phase. The two members in the pre-decision-making phase were in a social context of group decision making, but were not aware of each other’s choice preferences; at that time, their choice tendencies were influenced by social facilitation effects. By contrast, in the subsequent cooperative decision-making phase, in which both members made choices after indicating their respective decision preferences, the choice tendencies were influenced by information exchange. The group delay discounting rate was lower in the cooperative decision-making phase compared with that in the pre-decision-making phase, and the delayed option was preferred. This suggests that the group delay discounting rate is only influenced by the exchange of information among group members and is not solely dependent on the social facilitation effect of the presence of others.

#### 3.3.2. Information-Processing Processes

Bixter and Rogers [53] used videotaping to explore differences in information-processing processes between youth and elderly groups in group intertemporal decision making. Following Senecal et al. [66], they qualitatively coded the content of discussions between the two groups (the youth group and the elderly group) into four categories: perceived risk of the future, opportunity costs (e.g., interest rates, savings, investments), purchases or discussions of what the money could be spent on, and contrasting the monetary or delay attributes of the two rewards. The youth group and the elderly group both mentioned the value attributes or delay attributes of the two rewards the most during the group discussion, demonstrating that group intertemporal decision making was more inclined toward the attribute-based intertemporal choice model. However, compared with the youth group, the elderly group shared more information in the group discussion, which could have been because they had more life experience than the youth group. Nonetheless, the study did not analyze the relationship between information processing and group intertemporal decision making.

## 4. Future Research Prospects: Based on the “Two Processes” of Group Intertemporal Decision Making

As noted, research on group intertemporal decision making remains in the initial stages of exploration. Few studies address the differential mechanisms between group and individual intertemporal decision making or the mechanisms in group intertemporal decision-making processes. This paucity of research hinders the exploration of the theoretical mechanisms of group intertemporal decision making, as well as the development of strategies and policies for promoting more farsighted group intertemporal decisions. Building on the “process–outcome model” for assessing the quality of group decisions [61], future research could focus on the “two processes” of group intertemporal decision making to systematically reveal the psychological and theoretical mechanisms. At the level of interpersonal interaction, it could uncover the differential mechanisms between group and individual intertemporal decision making. At the level of information processing, it could elucidate the decision-making process mechanisms of group intertemporal decision making.

### 4.1. Mechanism of Differences between Group and Individual Intertemporal Decision Making in Terms of Interpersonal Interaction Processes

The most obvious difference between group decision making and individual decision making is that group decision making involves interactions among group members. Schwenke et al. [63] proposed that the interpersonal interaction process in group decision making includes two types of factors: information exchange and social facilitation. Thus, a question arises, as follows: would eliminating the information exchange process in group intertemporal decision making (e.g., by canceling the discussion process in group decision making) or adding social facilitation factors in individual intertemporal decision making (e.g., making individual decisions in the presence of others) reduce or eliminate the differences between group and individual intertemporal decision making? Future studies can adopt theoretical models of intertemporal decision making (e.g., the representative model, single-dimension priority) and manipulate the two types of factors in the interpersonal interaction process of group decision making. Then, they can measure and compare the decision-making processes of group and individual intertemporal decisions (e.g., examine the differences between dimensions) to reveal the mechanism of the differences between the two.

Most studies of interpersonal interaction processes in group decision making have focused on decision rules. Many studies have shown that the decision rules followed by the group affect group decision-making quality [46,67,68,69]. Studies typically focus on the unanimity rule and majority rule because they are more widely used in daily life [67,68]. Group decision making under the unanimity rule is more conducive to facilitating information exchange among group members and, therefore, has better decision quality than group decision making under the majority rule [70]. Future research on group intertemporal decision making can start at the level of decision rules, qualify the interpersonal interaction styles of group members, and explore how those styles differ under the unanimity rule and majority rule, thus affecting the final intertemporal decision-making outcome. Morone et al. [68], meanwhile, questioned the exploration of decision rules in the field of group decision making. They noted that, in most real-life situations, group members do not arrive at their decision outcomes under a predefined decision rule; rather, they tend to conduct group interactions and discussions based on decision rules derived from within the group. Therefore, the study explored group risk decision making without restricting the time spent on discussions and the way group members resolved their internal disagreements, ultimately exploring the rules of group risk decision making in a natural setting by observing and analyzing the group members’ discussion process. In the future, this experimental paradigm can also be used to explore group intertemporal decision making and the process of group intertemporal decision making in a natural setting.

Furthermore, when examining the interpersonal interaction process of group intertemporal decision making, in addition to applying a combination of traditional two-choice paradigms, titration paradigms, and self-assessment questionnaires, more novel approaches can be considered that allow the decision-making process to be directly observed. Schwenke et al. [63], for example, used visualization to measure group members’ intertemporal decision-making preferences. During group decision making, two participants controlled a cursor. One participant controlled the cursor’s vertical movement while the other controlled its horizontal movement. The movements of the two were superimposed so that the cursor would only begin to move if the two participants’ decision preferences were in agreement; if the cursor did not move, the two participants disagreed. This measure of intertemporal decision making allowed the group decision-making process to be visualized. Future research on group intertemporal decision making can further refine this visualization of intertemporal decision-making tasks and extract relatively objective indicators from them to record the group decision-making process (e.g., the direction in which group members move the cursor each time, the number of times group members conflict, and whether group members choose to compromise).

### 4.2. Mechanism of Group Intertemporal Decision Making in Terms of Information Processing

The group decision-making process is mostly recorded through audio and video recordings [3,61,71]. Speech length and frequency, as well as the viewpoints of group members, are coded and analyzed through peer assessment to explore information processing. However, existing studies rarely discuss the information-processing process. Bixter and Rogers [53] compared information processing in different age groups using video, but did not explore the connection between the information-processing process and decision-making outcomes. Therefore, future research can explore how to measure the breadth and depth of information processing in group intertemporal decision making and the factors that affect it (e.g., cognitive motivation and decision rules). It can also explore the relationships between the breadth and depth of information processing and the discount rate of group intertemporal decision making, as well as reveal the information-processing mechanism of group intertemporal decision making.

For instance, a recent study we conducted, based on motivated information-processing theory, manipulated time pressure during group decision making to induce different levels of cognitive motivation in the group. Using the video-aided peer review method, we coded and analyzed the group intertemporal decision-making process to examine the effects of cognitive motivation on the process, as well as the mediating roles of the depth and breadth of information processing. In the video-assisted peer evaluation method [61], the group intertemporal decision-making process can be recorded and transcribed to form a comprehensive repository of viewpoints. Subsequently, all viewpoints in the repository are categorized and discussed, dividing them into main categories, such as “dimension comparison” and “opportunity cost”. The “dimension comparison” category is further divided into subcategories such as “comparison of monetary dimensions,” “comparison of temporal dimensions”, and “comparison of differences in both monetary and temporal dimensions”. Thereafter, the depth and breadth of information processing under different decision-making conditions are calculated using a formula. This is used to explore the relationships between the depth and breadth of information processing and the group intertemporal decision discount rate, as well as reveal the mechanism of the group intertemporal decision-making process. This provides a basis for interventions to make group intertemporal decision making more farsighted.

The exchange of information between individuals can be likened to the exchange of information quanta in physical systems—a perspective that inspired Mindsponge Theory. Mindsponge Theory is an innovative theoretical framework that links microscopic quantum mechanics with macroscopic social psychology. According to Vuong [72], when information is thoroughly evaluated and deemed beneficial, it is integrated into our thinking patterns, becoming information that has core value or is highly trusted. This process is manifested at the human, biological, and even environmental levels, where the “brain” acts as a collector and processor of information, growing by filtering information from the surrounding environment. Vuong and Nguyen [73] further incorporated quantum physics into the interpretation of information and value, proposing the informational entropy-based notion of value. They argued that value is generated through information interaction, and information entropy not only quantifies the uncertainty of information, but also serves as a basis for measuring how individuals process and evaluate information. When the number of information units increases without a clear priority, information entropy increases, posing the risk of information loss. To reduce this risk, the brain expends energy to assess and integrate information units, forming deep-seated beliefs and preferences that aid individual decision-making and behavioral patterns (i.e., values). Future research can explore how individual values and information exchange between individuals affect group intertemporal decision making. By integrating social psychology with information technology, systems and tools that promote more efficient information exchange and decision making can be developed. These tools can enhance the efficiency of group decision making, especially in the face of global crises such as COVID-19, facilitating the assembly of collective wisdom and guiding more rational and sustainable decision making pathways. Through interdisciplinary research methods, we can gain an in-depth understanding of the psychological and theoretical mechanisms of group intertemporal decision making, providing robust guidance for practical decision-making practices.

## Data Availability

Not applicable.
